# In Patients Under Extracorporeal Co2 Removal Therapy (ECCO2R) For Ards Can We Do Prone Position? Efficiency, Stability and Safety of the Maneuver

**DOI:** 10.1186/2197-425X-3-S1-A511

**Published:** 2015-10-01

**Authors:** P Ngasseu, JP Ponthus, V Amilien, M Tchir, JF Georger

**Affiliations:** Centre Hospitalier Intercommunal de Villeneuve Saint Georges, Reanimation Polyvalente, Villeneuve Saint Georges, France; Centre Hospitalier Intercommunal de Villeneuve Saint Georges, Villeneuve Saint Georges, France

## Introduction

The mechanical ventilation of some patients with ARDS could be facility by ECCO2R allowing the reduction of blood acidosis and the reduction of tidal volume for the application of the protective ventilation. Prone position (PP) could be used for some patients with PaO2/FiO2 < 150. We don't know if we could associate PP and ECCO2R in ARDS patients

## Objectives

The aim of this study is to describe the feasibility of PP under ECCO2R, the stability of the parameters of the device and if we have side effect of the PP under ECCO2R.

## Methods

In our intensive care unit of 15 beds with a large experience of PP, we have retrospectively included all sessions of PP (at least 16 hours of PP) performed on patients under ECCO2R therapy between august 2014 and march.2015. We used ILA ACTIVVE^®^ device (NOVALUNG^®^) with MINILUNG^®^ membrane and a double line femoral catheter (NOVAPORT TWIN^®^ 24F). the gas flow was 10l/min. For each session we compared PaO2/FiO2 and the PaCO2 before and after 1H of PP. For each session, we did the mean of blood flow and drainage pressure (P1) during a length of one hour : during the last hour before PP, the first hour after PP and the last hour before stopping PP. We compared with a Friedman’s test, the mean and the coefficient of variation of each parameter to evaluate the stability of the device. We noted all the side effects of the PP (bleeding, decanulation, etc.).

## Results

We performed 9 PP sessions on 5 patients, 1 in 3 patients and 3 in 2 patients. the PaO2/FiO2 ratio was higher during PP (136(78-250) than before PP (126(58-145)). Between before, the beginning and the end of PP we didn't found difference in blood flow, respectively 1472ml/min (1201-1971), 1403ml/min (1216 - 1850), 1447ml/min (1231 - 2012), and in P1, respectively -37mmHg (-46- -25), -41mmHg (-50 - -28), -41mmHg (-47- -29).. the coefficient of variation of the blood flow was low and we didn't found variations of it between these 3 moments, respectively (0.9% (0.7 - 2.8), 0.7% (0.4- 2.1), 0.6% (0.4 - 1.6). the coefficient of variation of P1 was low and it was lower at PP than before PP(p < 0.05), respectively : 8.2% (3.7 - 9.9), 5.6% (2.8 - 6.8), 4.2% (2.9 - 5.8). We didn't found side effects of the PP maneuver.

## Conclusions

Prone position under ECCO2R with a femoral catheter is possible. We found no side effect of this technique. None difference in the blood flow, in the drainage pressure and in the stability of the blood flow were found. the stability of the drainage pressure is better in PP. the PaO2/FiO2 ratio is better on PP.

